# Clinical and public health implications of Supreme Court jurisprudence on gender-based violence: a qualitative analysis of Spanish rulings following organic law 10/2022

**DOI:** 10.3389/fpubh.2026.1850453

**Published:** 2026-09-23

**Authors:** David Casero-Benavente, Natalia Mudarra-García, Guillermo Charneco-Salguero, Leonor Cortes García-Rodríguez, Eva María García-Fernández, Cecilia Castillo-Gallardo, Francisco Lencina-Navarro, Inés Monedero Sánchez-Aranzueque, Ana Jiménez-Perianes, Francisco Javier García-Sánchez, José Miguel Cárdenas-Rebollo

**Affiliations:** 1 Nursing Department, University San Pablo-CEU, CEU Universities, Boadilla del Monte, Spain; 2 Instituto Ramón y Cajal de Investigación Sanitaria (IRYCIS), Madrid, Spain; 3 Health and Life Sciences Faculty, Universidad CEU-Fernando III, CEU Universities, Bormujos, Sevilla, Spain; 4 Psychology Department, University San Pablo-CEU, CEU Universities, Boadilla del Monte, Spain; 5 Emergency Room Service, IDIPHISA, Department of Medicine, University Complutense of Madrid, Hospital Universitario Infanta Cristina, Parla, Madrid, Spain; 6 Medical Department, Faculty of Medicine, University Complutense of Madrid, Madrid, Spain; 7 Department of Mathematics and Data Science, Faculty of Economics and Business Sciences, University San Pablo-CEU, CEU Universities, Madrid, Spain

**Keywords:** forensic evidence, gender-based violence, healthcare professionals, medico-legal documentation, public health

## Abstract

**Background:**

Gender-based violence (GBV) represents a major public health and legal challenge, requiring coordinated responses between healthcare and judicial systems. The implementation of Organic Law 10/2022 has introduced significant changes in the legal framework, influencing both judicial interpretation and clinical practice.

**Objective:**

To analyze interpretive patterns, evidentiary criteria, and jurisprudential trends in Spanish Supreme Court rulings on gender-based violence and habitual abuse, with particular attention to the role of healthcare professionals.

**Methods:**

A qualitative document analysis was conducted on Supreme Court rulings issued between 2023 and 2025. A total of 195 anonymized judgments were selected from the Aranzadi database. Data were analyzed using thematic coding, inductive categorization, and interpretative triangulation.

**Results:**

The victim's testimony has been consolidated as the principal form of evidence, provided it meets criteria of persistence, coherence, and credibility. The gender-based aggravating circumstance is applied within a contextual framework of domination. Sentence review under Organic Law 10/2022 follows the principle of favorable retroactivity, although interpretative inconsistencies persist. Habitual abuse is recognized as an autonomous offense requiring continuity, although its delimitation remains variable. Healthcare professionals play a key role in detection, documentation, and medico-legal support, contributing to evidentiary robustness.

**Conclusions:**

The findings highlight a progressive alignment between legal doctrine and healthcare practice. Strengthening clinical documentation, interdisciplinary coordination, and professional training is essential to improve both judicial outcomes and victim protection. This study underscores the critical role of healthcare professionals as evidentiary and preventive agents in gender-based violence.

## 1 Introduction

Gender-based violence (GBV) is a major global public health concern, with significant physical, psychological, and social consequences for affected individuals. The World Health Organization recognizes GBV as a priority issue requiring coordinated healthcare and societal responses, particularly due to its association with long-term morbidity, mental health disorders, and increased healthcare utilization (1). According to the most recent WHO global estimates, nearly one in three women worldwide (31.6%, approximately 840 million women) have experienced physical and/or sexual intimate partner violence or non-partner sexual violence at least once in their lifetime, a prevalence that has remained largely unchanged over the past two decades (2). In Spain specifically, official statistics from the Ministry of Equality report 1,368 femicide victims since national recording began in 2003, together with more than 2.6 million criminal complaints for gender-based violence filed between 2009 and 2025 and over 1.5 million calls to the national helpline (016) (3). These figures illustrate both the scale of the phenomenon and the central role that judicial and healthcare systems are called upon to play in its detection, documentation, and adjudication.

In Spain, GBV also represents a complex legal challenge. The jurisprudence of the Spanish Supreme Court plays a central role in defining interpretative criteria, particularly following the enactment of Organic Law 10/2022, which introduced substantial modifications in the regulation of sexual offenses and violence against women (4). This legislative reform has led to increased judicial activity, especially in the context of sentence review and evidentiary reassessment (5).

From a legal perspective, the assessment of evidence in GBV cases has traditionally focused on the victim's testimony, the concept of habitual abuse, and the application of aggravating circumstances such as gender or kinship (6, 7). However, these elements are not exclusively legal constructs; they are closely linked to clinical and psychosocial realities that are frequently first identified within healthcare settings.

Healthcare professionals, including physicians, nurses, and forensic specialists, play a crucial role in the early detection, documentation, and assessment of GBV. Clinical findings, psychological evaluations, and medical reports often constitute essential elements in the evidentiary process, reinforcing the credibility of the victim's testimony and contributing to judicial decision-making (8, 9). Moreover, healthcare settings frequently represent the first point of contact for victims, positioning clinicians as key agents in both prevention and intervention.

Despite this relevance, the interaction between healthcare-derived evidence and judicial interpretation remains insufficiently explored in the literature. Existing research has tended to examine legal criteria and clinical practice as separate domains: legal scholarship has concentrated on evidentiary standards, aggravating circumstances, and sentencing reform, while clinical and public health literature has focused on detection protocols, injury classification, and psychological assessment. To date, no study has systematically analyzed how Spanish Supreme Court jurisprudence – specifically following the entry into force of Organic Law 10/2022 – addresses the evidentiary weight of healthcare-derived information, leaving a gap in the understanding of how these two systems interact in practice. This gap is particularly significant given that Organic Law 10/2022 introduced a substantial reform of evidentiary and sentencing criteria whose real-world judicial application had not yet been qualitatively characterized.

Closing this gap matters for both fields. From a clinical and public health perspective, understanding how healthcare-related evidence is interpreted and weighted in judicial settings is essential to improve detection, documentation, and interdisciplinary management of GBV, and may inform more effective clinical protocols and professional training. From a legal perspective, characterizing how courts have applied the reformed evidentiary and sentencing framework provides an empirical basis for identifying interpretative inconsistencies that affect legal certainty and victim protection. Addressing both dimensions jointly, rather than separately, is precisely the contribution this study aims to make.

Therefore, the main objective of this study is to conduct a qualitative analysis of Spanish Supreme Court judgments related to GBV issued after the entry into force of Organic Law 10/2022. The secondary objectives are to identify interpretative patterns and jurisprudential trends, to characterize the evidentiary criteria applied by the Court, and to evaluate the role of healthcare professionals as evidentiary and preventive agents within this judicial context.

## 2 Materials and methods

### 2.1 Study design

This study follows a qualitative research design based on document analysis, using content analysis techniques applied to judicial texts.

### 2.2 Data source and collection

The corpus consisted of rulings issued by the Spanish Supreme Court, Second Chamber (Sala de lo Penal), in the field of criminal law, specifically related to gender-based violence, habitual abuse, and related offenses. Data were collected through the Aranzadi Instituciones database (Thomson Reuters), accessed under institutional library subscription of University San Pablo-CEU, which grants authorized academic users access to its repository of published, anonymized case law for research purposes.

Retrieval combined thematic and Boolean date-filtered searches within the database's criminal-law repository, restricted to the Supreme Court and to the period between 1 January 2023 and 31 December 2025. Search terms/filters used were “violencia de género” AND “malos tratos habituales”. Searches were conducted independently by two reviewers, and discrepancies in the resulting record set were resolved by a third reviewer.

### 2.3 Inclusion and exclusion criteria

The inclusion criteria were as follows:

- Supreme Court rulings in the criminal jurisdiction.
- Cases related to domestic or gender-based violence.
- Judgments issued between 2023 and 2025.
- Rulings aligned with the legal framework of Organic Law 10/2022.
- Full-text rulings available in the Aranzadi Instituciones database.
- Rulings resolving an appeal in cassation (*recurso de casaciǿn*) on the substantive merits of a gender-based violence or habitual abuse charge.

The exclusion criteria were:

- Judgments from lower courts (provincial or national courts).
- Cases prior to 2023.
- Cases outside the scope of family or gender-based violence.
- Non-anonymized or restricted-access rulings.
- Purely procedural or admissibility decisions not addressing the substantive merits of the case.
- Rulings with incomplete or corrupted text preventing thematic coding.

### 2.4 Sample selection

An initial dataset of 105,067 criminal law rulings was identified. After filtering for Supreme Court decisions, 1,663 rulings were obtained. Applying the temporal and thematic criteria, 195 rulings were included in the final qualitative analysis. No duplicate cases were identified (Figure 1).

**Figure 1:**
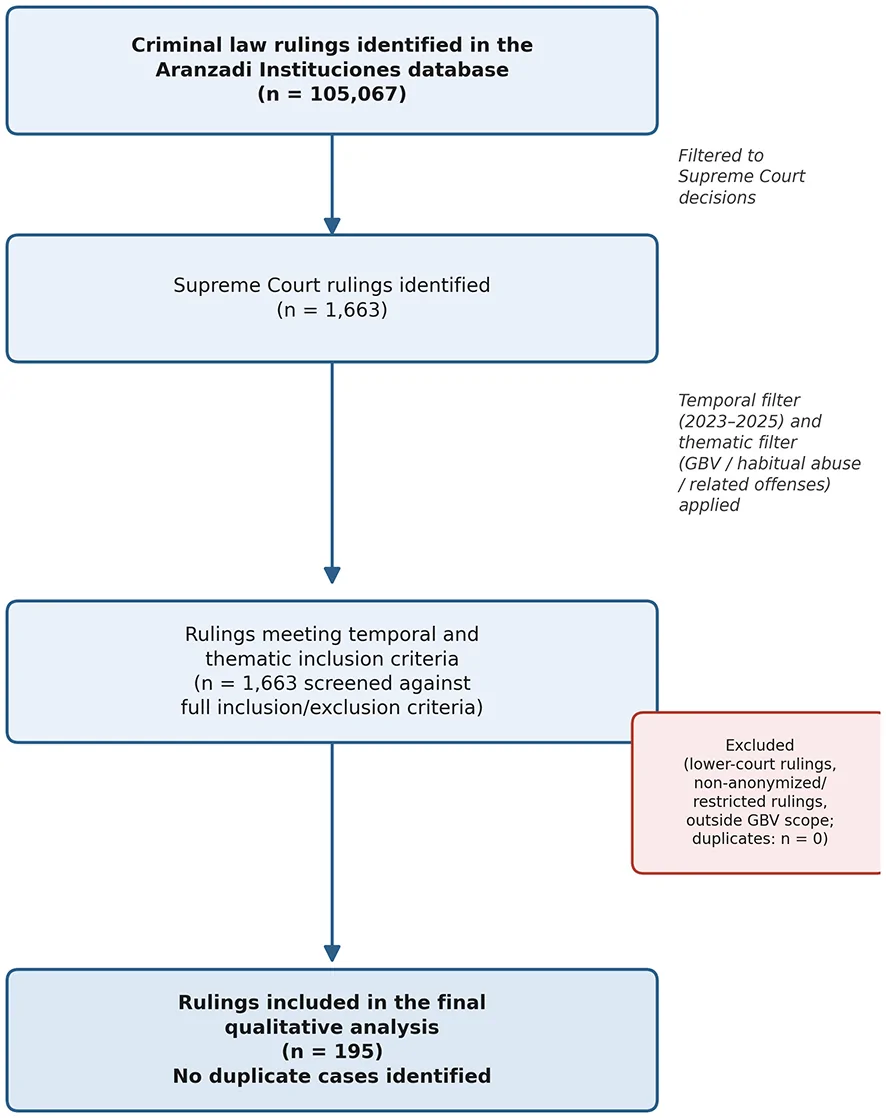
Flow diagram of the study selection process of Supreme Court rulings included in the qualitative analysis.

### 2.5 Data analysis

The analytical process was conducted in three stages:

- **Thematic coding**: a coding matrix was developed, including categories such as evidentiary assessment, aggravating and mitigating factors, sentence review, and the role of healthcare professionals.
- **Inductive categorization**: categories were derived directly from the judicial texts without pre-defined assumptions.
- **Interpretative triangulation**: the legal reasoning of the rulings was contrasted with judicial outcomes, legislative developments, and healthcare-related literature.

Qualitative analysis was performed using Atlas.ti software. SPSS version 29 was used for descriptive statistical support of coded frequencies.

Reliability of the thematic coding was addressed through interpretative triangulation and joint discussion among the review team, as described above, rather than through a separately quantified inter-coder agreement statistic.

### 2.6 Use of AI tools

AI-based tools (Copilot and Gemini) were used exclusively to support linguistic structuring, legal comprehension, and technical revision of the manuscript. These tools did not intervene in data interpretation or analytical processes.

### 2.7 Ethical considerations

This study was based exclusively on judicial rulings that are publicly available in anonymized form through the Aranzadi Instituciones database; no primary data collection involving human participants, patients, or victims was undertaken, and no identifiable personal information was accessed or processed at any stage. In accordance with University CEU San Pablo's research ethics policy, secondary analyses of publicly available, anonymized documentary sources of this kind do not require formal ethics committee review, since they do not involve identifiable individuals, biological samples, or any intervention. No personal or identifiable data were used at any stage of data collection, coding, or analysis.

## 3 Results

### 3.1 Distribution of thematic dimensions

A total of 2,940 coded mentions were identified across the analyzed rulings. The legal dimension accounted for 85.14% of the total, whereas the healthcare-related dimension represented 14.86% (Table 1).

**Table 1:** Distribution of frequencies and percentage weights of coded mentions in Supreme Court judgments (*N* = 2,940 mentions across 195 rulings).

Dimension / category	Mentions (f)	Percentage (%)
**Legal dimension**	**2,503**	**85.14**
Continuous patterns / verbal violence	502	17.08
Other offenses (assaults, threats, etc.)	640	21.77
Application of aggravating factors	357	12.14
Habitual abuse (autonomous offense)	355	12.08
Domestic violence (general)	260	8.84
Gender-based violence (no reduction under LO 10/2022)	240	8.16
Appeals in cassation	149	5.07
**Healthcare dimension**	**437**	**14.86**
Other healthcare/preventive indicators	388	13.20
Forensic psychological evaluation	26	0.88
Clinical intervention and protocols	23	0.78
**Total**	**2,940**	**100.00**

Bold values indicate total frequencies and percentages of coded mentions across all dimensions.

Within the legal dimension, the most frequently identified categories included continuous patterns of violence (17.08%), application of aggravating factors (12.14%), habitual abuse as an autonomous offense (12.08%), and general domestic violence (8.84%). Other categories, such as assaults, threats, and related offenses, accounted for 21.77% (Table 2).

**Table 2:** Distribution of types of violence in Supreme Court judgments.

Type of violence	Mentions (f)	Percentage (%)
Habitual abuse (continuous pattern)	355	12.08
Domestic violence (family context)	260	8.84
Verbal and psychological violence	251	8.54
Physical violence (assault)	48	1.63
Threats (serious and minor)	89	3.03
Sexual violence / rape	34	1.16

Within the healthcare dimension, most references corresponded to general preventive or clinical indicators (13.20%), while specific references to clinical intervention (0.78%) and forensic psychological evaluation (0.88%) were less frequent (Figure 2).

**Figure 2:**
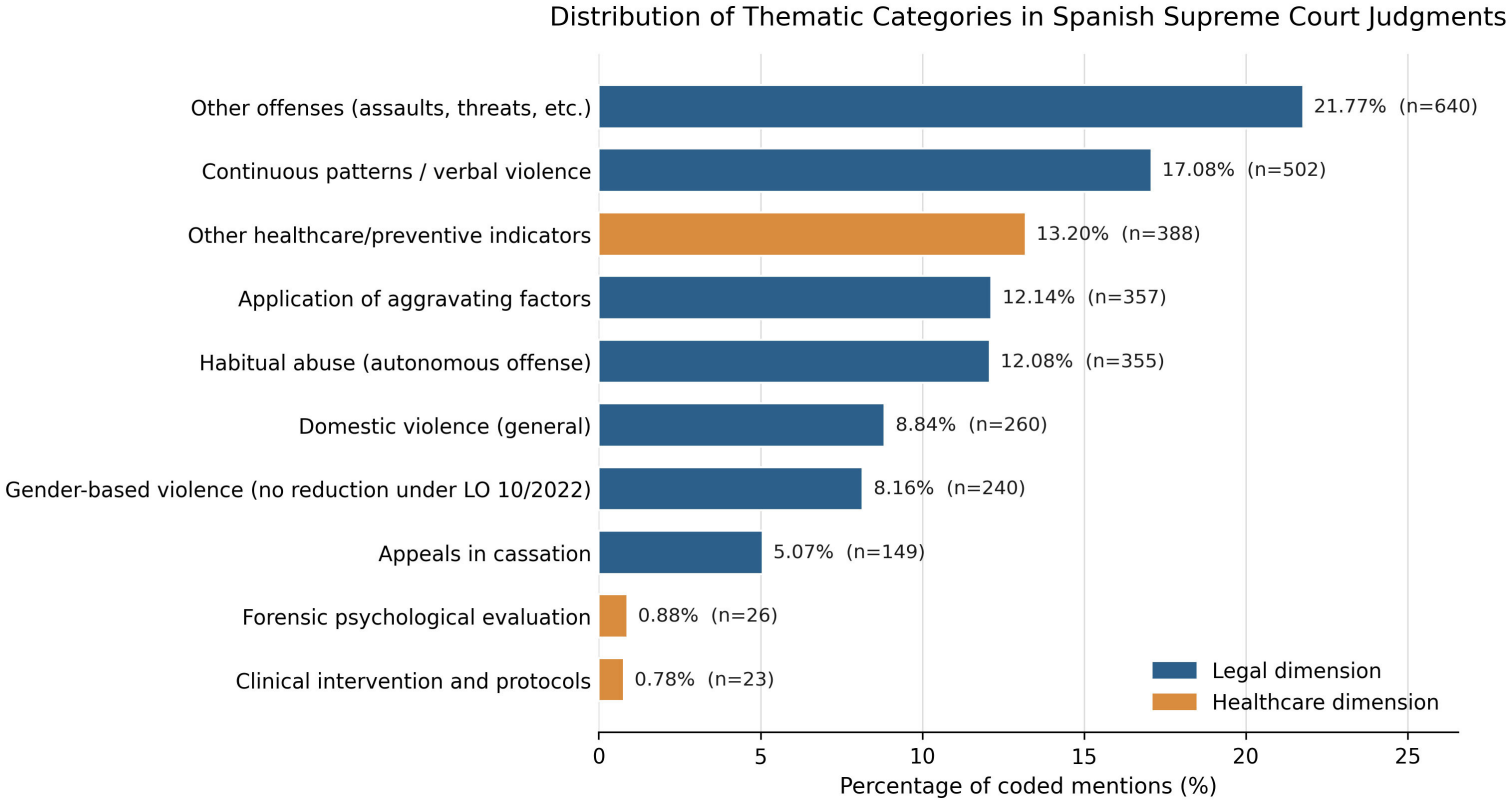
Distribution of the main thematic categories identified in Spanish Supreme Court judgments included in the qualitative analysis.

### 3.2 Evidentiary assessment

The victim's testimony was consistently identified as the primary form of evidence, provided it met three criteria: persistence, coherence, and absence of subjective incredibility. Courts required enhanced reasoning when convictions relied exclusively on the victim's statement.

*Example:* “The Court considers that the victim's statement, consistently maintained since the investigative phase, is credible and sufficient to support the conviction.”

### 3.3 Aggravating and mitigating factors

The gender-based aggravating factor was applied systematically but not automatically. Its application required the existence of a context of domination or discrimination based on gender. The aggravating factor of kinship was limited to cases involving stable cohabitation.

*Example:* “The defendant's conduct is framed within a context of domination and control over the victim, which justifies the application of the gender-based aggravating factor.”

### 3.4 Sentence review under organic law 10/2022

A consistent application of the principle of favorable retroactivity was observed. Sentence review focused on the adjustment of penalties rather than on the reassessment of guilt. Appeals were pre-dominantly based on disagreements regarding evidentiary evaluation.

*Example:* “The review of the imposed sentence is appropriate, as the new sentencing framework established by Organic Law 10/2022 is more favorable.”

### 3.5 Habitual abuse

Habitual abuse was identified as an autonomous criminal offense requiring repetition and continuity over time. Courts clearly distinguished between isolated incidents and sustained patterns of violence.

*Example:* “Multiple episodes of violence over a two-year period have been confirmed, establishing habitual abuse.”

### 3.6 Appeals and judicial outcomes

Most appeals were based on the assessment of evidence. The overall dismissal rate was 57.95%, indicating that in the majority of cases, the Court upheld the reasoning of the lower courts. The combined rate of full and partial success was 15.39%, with partial estimations being more frequent than full ones (Figure 3).

**Figure 3:**
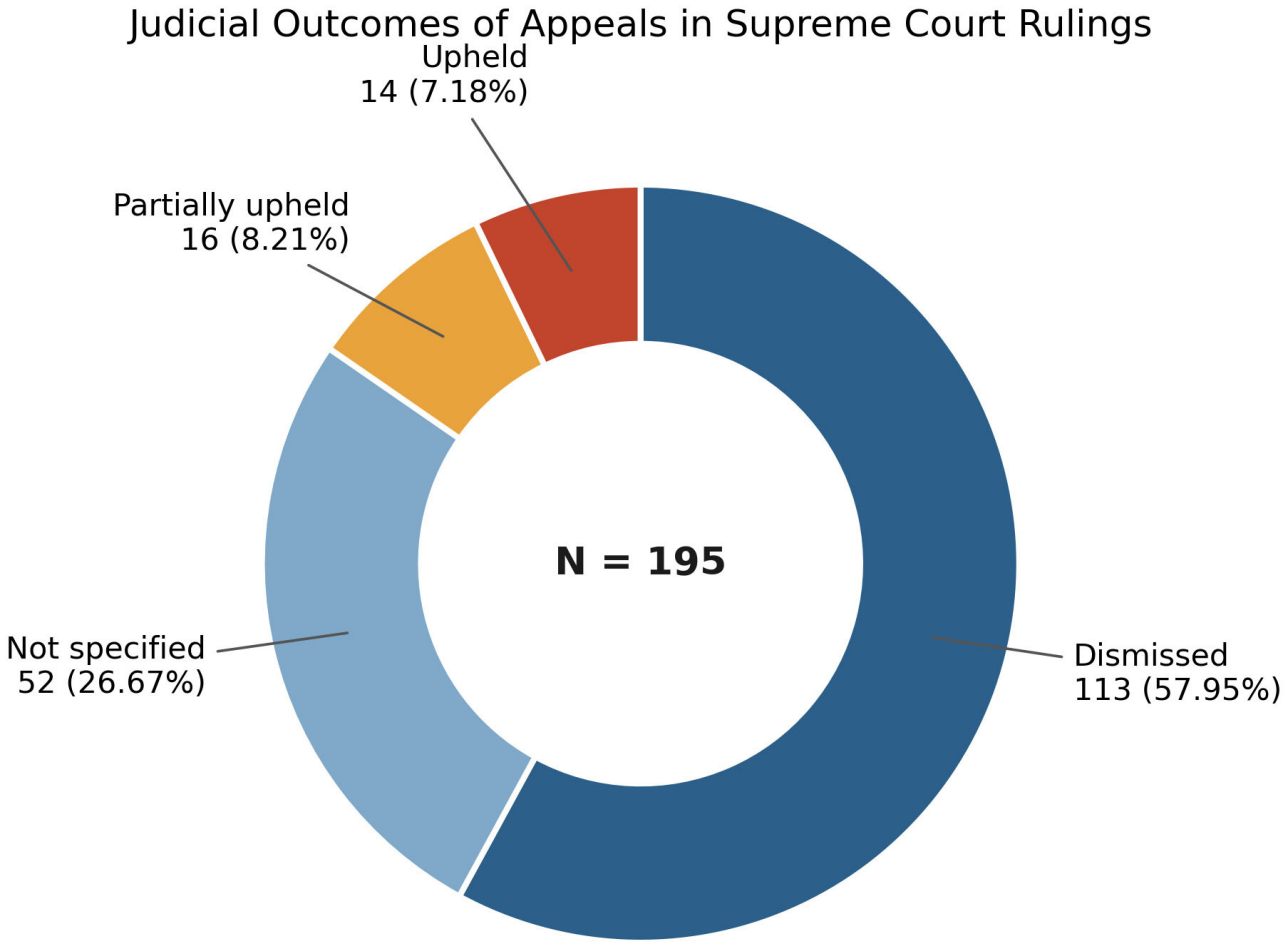
Judicial outcomes of appeals in Supreme Court rulings.

These findings suggest a strong doctrinal consolidation, with limited variability in the interpretation of evidentiary standards (Table 3).

**Table 3:** Statistical analysis of judicial outcomes in Supreme Court rulings (*N* = 195).

Outcome of appeal	Frequency	Percentage (%)
Dismissed	113	57.95
Not specified	52	26.67
Partially upheld	16	8.21
Upheld	14	7.18
**Total**	**195**	**100.00**

Bold values represent the total number of cases (N) and cumulative percentages of judicial outcomes.

### 3.7 Role of healthcare professionals

Healthcare professionals were identified as relevant evidentiary agents, particularly through clinical documentation and forensic reports. Their contributions reinforced the credibility of the victim's testimony and supported the classification of habitual abuse (Table 4).

**Table 4:** Legal–healthcare coding matrix.

Dimension	Category	Subcategory	Coding criteria
Legal	Evidentiary assessment	Victim testimony	Persistence, coherence, absence of subjective incredibility
		External witnesses	Testimonies corroborating the victim's account
		Documentary evidence	Medical reports, prior complaints, messages
Legal	Aggravating factors	Gender	Context of domination or discrimination
		Kinship	Stable relationship or cohabitation
		Recidivism	Previous similar offenses
Legal	Sentence review	Favorable retroactivity	Application of more beneficial legal framework
		Limits of review	Affects sentencing but not guilt
Legal	Habitual abuse	Temporal repetition	Repeated violent episodes over time
		Continuity	Patterned behavior vs. isolated events
Healthcare	Clinical intervention	Early detection	Identification of violence indicators
		Medical report	Clinical assessment of injuries
		Psychological evaluation	PTSD or related symptoms
Healthcare	Coordination	Judicial referral	Submission of reports to legal authorities
		Court participation	Expert witness role
Healthcare	Ethics	Confidentiality vs. reporting	Ethical dilemmas in clinical practice
Healthcare	Training	Professional education	Training in GBV protocols

Although less frequently represented than legal elements, healthcare-related contributions were particularly relevant in cases lacking direct witnesses or material evidence.

## 4 Discussion

The present qualitative analysis of Spanish Supreme Court case law on gender-based violence and habitual abuse following the enactment of Organic Law 10/2022 provides relevant insights into the current legal–clinical interface and its implications for healthcare practice (4, 10).

### 4.1 Consolidation of evidentiary criteria and implications for clinical practice

One of the most consistent findings is the doctrinal consolidation of the victim's testimony as the principal form of evidence, provided that it meets the criteria of persistence, coherence, and absence of subjective incredibility. This aligns with previous legal scholarship highlighting the progressive strengthening of testimonial evidence in gender-based violence cases (6, 11).

From a clinical perspective, this evolution has direct implications. The increasing reliance on the victim's account places greater importance on the role of healthcare professionals in documenting clinical findings that support or contextualize such testimony. Medical records, psychological evaluations, and longitudinal observations may act as reinforcing elements that enhance judicial confidence in the victim's narrative, particularly in the absence of direct witnesses or material evidence.

Furthermore, the requirement for enhanced judicial reasoning when testimony is the sole evidence introduces an additional layer of rigor that indirectly demands higher quality and consistency in clinical documentation, reinforcing the medico-legal value of healthcare interventions.

### 4.2 Application of gender-based aggravating circumstances

The findings also confirm a structured, although non-automatic, application of the gender-based aggravating circumstance, which requires the presence of a context of domination or discrimination. This interpretation has been positively evaluated in doctrinal literature (7), as it avoids purely formal application and aligns with constitutional guaranties and international standards on gender-based violence.

However, variability in its application persists, as previously described (12, 13). This inconsistency has relevant implications for both legal and clinical settings. In particular, healthcare professionals may be required to identify and describe contextual elements of domination, control, or coercion that go beyond physical injury and involve psychosocial and behavioral (14).

This reinforces the need for a bio-psychosocial assessment approach in clinical practice, where contextual factors are systematically recorded and integrated into medico-legal reports.

### 4.3 Sentence review and legal uncertainty

The application of the principle of favorable retroactivity under Organic Law 10/2022 reflects a consistent judicial effort to adapt sentencing to the updated legal framework (15). Nevertheless, interpretative discrepancies remain, particularly in borderline cases, as noted in the literature (16).

These inconsistencies generate a degree of legal uncertainty that may indirectly affect clinical practice. Healthcare professionals involved in forensic or expert assessments may face challenges when their reports are interpreted under evolving legal criteria. Therefore, closer alignment between legal standards and clinical reporting frameworks is required to ensure consistency and reliability.

### 4.4 Habitual abuse: conceptual and practical challenges

Habitual abuse is consistently recognized as an autonomous criminal offense requiring repetition and continuity. However, the absence of clear temporal or quantitative thresholds continues to generate interpretative variability (7, 17).

From a healthcare perspective, this ambiguity underscores the importance of longitudinal clinical documentation. Repeated consultations, patterns of injury, psychological symptoms, and behavioral indicators may collectively contribute to establishing the continuity required for legal classification (18).

This finding reinforces the role of healthcare professionals not only as providers of acute care but also as key contributors to the identification of chronic and recurrent patterns of abuse.

### 4.5 Critical perspectives on evidentiary centrality

Despite the positive evolution of evidentiary standards, concerns persist regarding the potential over-reliance on testimonial evidence. Some authors have highlighted the risk of a de facto shift in the burden of proof in cases lacking objective corroboration (9).

This issue has important ethical and procedural implications. While strengthening victim protection is essential, it must be balanced with the preservation of procedural guaranties, including the presumption of innocence. In this context, the contribution of healthcare professionals becomes even more critical, as objective clinical evidence may help mitigate this tension, providing additional support without replacing judicial (19).

### 4.6 International perspective: healthcare–judicial integration models

Comparison with other European jurisdictions helps situate these findings within a broader context. In Italy, the introduction of a dedicated emergency department pathway for victims of violence – the so-called “pink code” (*codice rosa*) – has institutionalized the early involvement of a multidisciplinary team, including a forensic physician, from the moment a victim presents at the emergency department, with the explicit aim of improving both clinical care and the evidentiary value of forensic documentation (20). Under this model, the forensic physician's involvement is not contingent on the case being perceived as severe but is built into the standard care pathway, which the literature identifies as key to preventing the loss of transient forensic evidence and producing more legally robust documentation (20).

By contrast, the Supreme Court rulings analyzed in the present study suggest a less formalized integration: healthcare-derived evidence appears in the judicial record pre-dominantly through *ad hoc* medical reports and expert testimony rather than through a standardized, institutionalized clinical-forensic pathway comparable to the pink code. This is consistent with prior medico-legal literature indicating that the Spanish context, similarly to other Southern European countries, still faces challenges in the systematic articulation between emergency healthcare response and forensic-judicial documentation (20). This comparison suggests that a standardized, institutionally embedded clinical-forensic pathway – rather than case-by-case referral – could further strengthen the evidentiary robustness that Spanish Supreme Court jurisprudence increasingly demands of healthcare-derived documentation.

### 4.7 The role of healthcare professionals in the judicial process

The findings confirm the central role of healthcare professionals in the evidentiary construction of gender-based violence cases, as previously described in forensic and clinical literature (5, 8).

Their contribution extends across multiple domains:

- Detection of early signs of violence.
- Documentation of physical and psychological harm.
- Expert assessment in forensic and judicial contexts.
- Activation of protection mechanisms.

In particular, psychological and medical reports are frequently used as complementary evidence that reinforces the credibility of the victim's testimony. This highlights the need for standardized protocols and structured reporting formats to ensure that clinical information is both accurate and legally relevant.

This evidentiary role is most effectively exercised through close collaboration between treating clinicians and forensic physicians, whose combined expertise bridges the therapeutic and medico-legal dimensions of care. Treating clinicians are typically best positioned to identify and document injuries and clinical indicators at first contact, while forensic physicians contribute the objective, legally oriented rigor – injury characterization, causal-nexus analysis, and chain-of-custody procedures – that subsequent judicial proceedings require (20). Strengthening formal channels for this clinical-forensic collaboration, rather than treating it as an occasional consultation, is likely to improve both the completeness and the judicial usability of the evidentiary record.

It is also important to recognize that this evidentiary and documentation responsibility represents an additional demand on healthcare professionals who already operate under considerable occupational strain. Violence and aggression directed at healthcare and social-care workers themselves are a well-documented and growing concern across Western health systems (21), and the added burden of medico-legal documentation, expert testimony, and coordination with judicial authorities in GBV cases compounds an already demanding workload. Acknowledging this burden is not a reason to diminish the evidentiary role of healthcare professionals in GBV cases, but a reason to ensure that adequate time, training, institutional support, and protection are provided so this role can be sustained without contributing to professional burnout.

### 4.8 Ethical and training-related challenges

The study also identifies relevant ethical dilemmas, particularly regarding the balance between confidentiality and the duty to report situations of risk. Additionally, variability in training and experience among healthcare professionals may limit the quality and utility of clinical reports in judicial proceedings.

These findings are consistent with international recommendations, including those of the World Health Organization (22), which emphasize the need for specialized training and institutional support in the management of gender-based violence.

Improving training, consultation time, and interdisciplinary coordination is, therefore, essential to enhance both clinical care and medico-legal effectiveness.

Beyond individual variability in professional training, the persistence of a gender gap in academic and forensic medicine leadership may further compound these institutional limitations. Recent narrative reviews on the academic forensic medicine field have documented that, despite the increasing representation of women among practicing physicians, women remain markedly underrepresented in senior academic and leadership roles, including the directorship of forensic medicine residency programs, where fewer than 20% of Italian programs are led by women (23). Insofar as academic leadership shapes curricular priorities, training protocols, and the institutional allocation of resources for specialized education on gender-based violence, this imbalance may indirectly affect the consistency and depth of the training available to the healthcare professionals who participate in the evidentiary process described above. Addressing structural gender disparities within academic medicine itself may therefore represent a complementary, upstream strategy for strengthening the clinical-forensic training pipeline.

### 4.9 Strengths and contribution of the study

This study provides a novel qualitative perspective by directly analyzing Supreme Court rulings and integrating legal and healthcare frameworks. It contributes to bridging the gap between clinical practice and judicial interpretation, offering insights that may inform both policy and practice.

At the same time, it highlights the need for further empirical research integrating clinical data and judicial outcomes, particularly in comparative international contexts.

### 4.10 Limitations

This study has several limitations that should be considered when interpreting its findings. First, the corpus was drawn exclusively from the Aranzadi Instituciones database; although this is one of the most comprehensive legal databases available in Spain, rulings not indexed in this specific repository could not be captured, which may affect the completeness of the sample. Second, this is a retrospective analysis of published judgment texts rather than the underlying clinical or forensic records themselves; the study reflects how healthcare-derived evidence is represented and weighted *within* judicial texts, not the completeness or quality of the original clinical documentation. Third, the two-year observation window (2023–2025), while appropriate for capturing early jurisprudential application of Organic Law 10/2022, may not be sufficient to detect longer-term interpretative trends. Fourth, although thematic coding was developed through inductive categorization and interpretative triangulation, qualitative coding inherently involves interpretive judgment. Fifth, the study is confined to the Spanish legal and healthcare context; the interpretative patterns identified may not generalize to jurisdictions with different evidentiary standards or health-system structures. Finally, AI-based tools were used exclusively for linguistic and structural support during manuscript preparation and did not participate in data interpretation or analysis; their use in language editing is nonetheless disclosed for full transparency.

### 4.11 Implications for clinical practice and future research

Overall, the results highlight a progressive alignment between legal doctrine and clinical practice, although important gaps remain. The consolidation of interpretative criteria represents a positive development, but it must be accompanied by:

- Improved clinical documentation standards.
- Enhanced training in gender-based violence.
- Greater integration between healthcare and judicial systems.
- Development of unified medico-legal protocols

Future research should focus on comparative and longitudinal analyzes to assess the real-world impact of legislative reforms on both patient outcomes and judicial processes, as well as on the effectiveness of healthcare-based interventions in improving evidentiary quality and victim protection.

## 5 Conclusions

This study provides a qualitative analysis of Spanish Supreme Court jurisprudence on gender-based violence and habitual abuse following the enactment of Organic Law 10/2022, identifying key interpretative patterns and evidentiary criteria.

First, the victim's testimony has been consolidated as the primary form of evidence, provided that it meets the established criteria of persistence, coherence, and credibility. This reinforces judicial protection mechanisms, although it requires enhanced judicial reasoning to ensure procedural guaranties.

Second, the application of the gender-based aggravating factor shows a trend toward a more precise and contextualized interpretation, requiring evidence of domination or discrimination. However, challenges remain in the consistent delimitation and justification of its application.

Third, the application of the principle of favorable retroactivity has allowed the adaptation of sentences to the new legal framework. Nevertheless, interpretative discrepancies persist in borderline cases, generating a degree of legal uncertainty.

Fourth, habitual abuse is recognized as an autonomous criminal offense requiring repetition and continuity over time. However, the absence of clear temporal and quantitative thresholds may lead to variable interpretations.

Fifth, the findings highlight the essential role of healthcare professionals as both evidentiary and preventive agents. Clinical documentation and forensic evaluation contribute significantly to reinforcing the credibility of victim testimony and to the classification of abuse patterns.

Finally, the study underscores the importance of strengthening interdisciplinary collaboration between healthcare and legal systems. Future strategies should prioritize the development of standardized protocols, improved professional training, and integrated approaches to enhance both clinical practice and judicial responses to gender-based violence.

## Data Availability

The original contributions presented in the study are included in the article/supplementary material, further inquiries can be directed to the corresponding author.
